# Investing in Family Planning: Key to Achieving the Sustainable Development Goals

**DOI:** 10.9745/GHSP-D-15-00374

**Published:** 2016-06-20

**Authors:** Ellen Starbird, Maureen Norton, Rachel Marcus

**Affiliations:** a U.S. Agency for International Development, Washington, DC, USA

## Abstract

Voluntary family planning brings transformational benefits to women, families, communities, and countries. Investing in family planning is a development “best buy” that can accelerate achievement across the 5 Sustainable Development Goal themes of People, Planet, Prosperity, Peace, and Partnership.

## INTRODUCTION

Family planning encompasses the services, policies, information, attitudes, practices, and commodities, including contraceptives, that give women, men, couples, and adolescents the ability to avoid unintended pregnancy and choose whether and/or when to have a child. In this commentary, we outline family planning’s links to the Sustainable Development Goals (SDGs) and highlight the transformational benefits that voluntary family planning brings to women, families, communities, and countries. We present family planning as a cross-sectoral intervention that can hasten progress across the 5 SDG themes of People, Planet, Prosperity, Peace, and Partnership (Figure). We particularly stress family planning’s:

- Link to human rights, gender equality, and empowerment
- Impact on maternal, newborn, child, and adolescent health
- Role in shaping economic development and environmental and political futures

**FIGURE:**
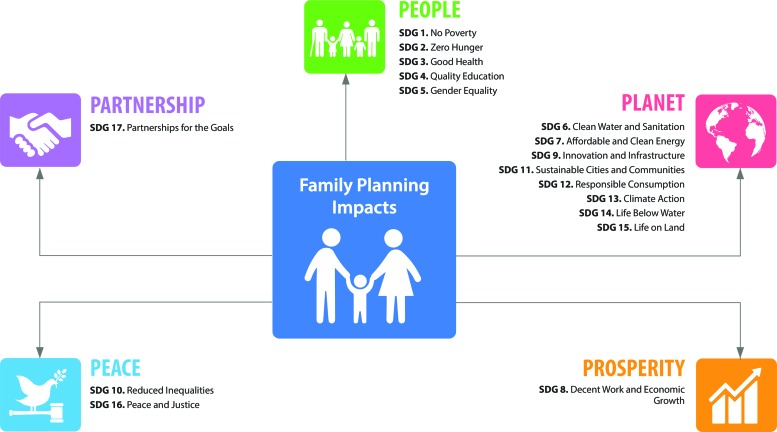
The 5 Sustainable Development Goal Themes of People, Planet, Prosperity, Peace, and Partnership

Accelerating progress in these areas is critical for SDG achievement.

We set forth evidence on ways that family planning can influence SDG achievement. At times, the evidence is strong; at other times, less so. Our hope is that the evidence gaps will motivate researchers to address unanswered questions. Most importantly, we hope that the evidence presented here leads to action at the international and country level—to fully support organized, voluntary family planning in the public and private commercial sectors, as well as through civil society.

This paper outlines the multiple reasons why investing in family planning is a good decision at every level. It is aligned with recent studies that find that investing in family planning is a development “best buy.”^1^ Accordingly, we hope that the information presented here will help governments and planners—including Ministries of Finance, district health teams, and civil society organizations—to consider family planning as a fundamental element of any long-term, socioeconomic development strategy, and key to SDG achievement.

Investing in family planning is a development “best buy.”

In 2000, representatives from 189 United Nations Member States endorsed 8 Millennium Development Goals (MDGs) to be achieved by 2015, and affirmed their collective commitment to poverty reduction and improved quality of life. However, during the next decade, progress toward MDG 4 (reduce child mortality), 5 (improve maternal health), and 6 (combat HIV/AIDs, malaria, and other diseases) was relatively slow. In fact, MDG 5.B, universal access to reproductive health, including access to voluntary family planning (not added until 2007), witnessed the least progress over the entire 15-year MDG time frame. By 2010, experts concluded that “the poorest, least educated women in sub-Saharan Africa have lost ground, with adolescents lagging farthest behind.”^2^

The international community, however, has made important strides in recent years. The 2010 “Global Strategy for Women’s and Children’s Health” has mobilized new resource commitments, and Family Planning 2020 (FP2020), the UN Commission on Life-Saving Commodities, the MDG Health Alliance, and other groups have revitalized family planning globally. Civil society organizations are highly engaged at local levels to ensure the positive momentum continues. Despite this renewed momentum, family planning investments and service access fall short of need in virtually all low-resource settings.

Despite renewed momentum, family planning investments and service access fall short of need in virtually all low-resource settings.

Below, we present the SDGs using the organizing principles set forth in the preamble of the Sustainable Development Goals—People, Planet, Prosperity, Peace, and Partnership. (Thus, the SDGs are not always presented in numerical order in this article.) We then synthesize the most recent analyses that document family planning’s importance for the achievement of the SDGs.

## PEOPLE

Family planning affects people in myriad ways. Most fundamentally, it advances human rights.

Voluntary family planning helps women and men secure their rights to decide freely, and for themselves, whether, when, and how many children they want to have—a basic human right.^3^ Family planning supports the rights of the girl child to remain unmarried and childless, until she is physically, psychologically, and economically ready, and desires to bear children. It supports the rights of adolescent boys and girls to information on how rapid, repeat pregnancies will affect their future. It strengthens the rights of women with HIV to decide on future childbearing, free of coercion. Family planning supports the rights of all people to accurate, unbiased information on contraceptive methods that can help them achieve their reproductive preferences. Yet, in many countries, despite possessing these inherent rights, women and girls often bear more children than they want, or at times when they are not planned.

In 2012, the year for which the most recent data are available, approximately 85 million pregnancies, representing 40% of all pregnancies globally, were unintended.^125^ This number was projected to rise to 92 million by 2015.^4^ In 2014, 225 million women in the developing world had an unmet need for a modern contraceptive method.^5^ Women with unmet need are defined as those who want to stop or delay childbearing but are not using modern contraceptive methods.

In 2012, approximately 85 million pregnancies were unintended.

SDGs 3.7 and 5.6 support “universal access to sexual and reproductive health-care services, including for family planning” and “universal access to sexual and reproductive health and reproductive rights,” respectively.^6^

SDG 3.7 supports universal access to sexual and reproductive health care services, including family planning. SDG 5.6 supports universal access to sexual and reproductive rights.

Beyond human rights, family planning affects people in other ways, as outlined below.

### Goal 1. No Poverty: End Poverty in All Its Forms Everywhere

#### Family Planning Helps Reduce Poverty

Over the last 3 decades, extreme poverty has declined significantly. In 1981, 50% of the developing world’s population lived on less than US$1.25 per day. In 2010, this indicator had dropped to 21%.^7^ While population’s links to poverty have been debated over the years, a consensus is emerging that rapid population growth can increase the sheer number of poor people.^8^

- The latest data show that the share of Africans who were poor fell from 56% in 1990 to 43% in 2012. Yet, due to population growth, many more people are poor—about 330 million in 2012, up from about 280 million in 1990.^9^
- Equally important, African population growth is not slowing as quickly as anticipated. In 2015, the UN estimated that Africa had the highest annual population growth rate among major geographic areas (2.55%) and projected that it would remain high in 27 African countries.^10^
- Between 2015 and 2050, an estimated 1.3 billion people will be added in Africa. The populations of Angola, Burundi, Democratic Republic of the Congo, Malawi, Mali, Niger, Somalia, Uganda, United Republic of Tanzania, and Zambia may increase at least fivefold by 2100.^10^

The 2015 UN report of population estimates and projections concludes, “… population growth in the poorest countries will make it harder for those governments to eradicate poverty and inequality … [and] improve the provision of basic services.”^10^ The challenges for poverty reduction strategies and family planning are clear.

At the household level, some studies caution against “the widely held view that large families are poorer” and fail to find links between household size and poverty.^11^ Other studies take a different approach to examining family planning’s contribution to poverty reduction. They focus instead on family planning’s role in creating human capital. A 2010 study found that the family planning program in Colombia reduced women’s completed lifetime fertility by approximately one-half of a child and explained a relatively low 6% to 7% of the fertility decline between 1964 and 1993.^12^ “Despite its modest role in reducing lifetime fertility,” the study concluded, “the ability of family planning to fight poverty cannot be easily dismissed.” The study found that women with access to family planning as teenagers gained 0.05 more years of schooling, were 7% more likely to work in the formal sector, and were 2% less likely to cohabit with male partners. In addition, young Colombian women with access to modern contraception “experienced substantial socio-economic gains” because contraception allowed them to postpone their first births and determine their life course. The study concluded that these estimates may place family planning “among the most effective (and cost-effective) interventions to foster human development.”^12^

This work links to other SDGs related to economic development and poverty reduction, including Goal 8 (decent work and economic growth) and Goal 10 (reduced inequalities).

### Goal 2. Zero Hunger: End Hunger, Achieve Food Security and Improved Nutrition, and Promote Sustainable Agriculture

#### Family Planning Contributes to Improved Nutrition Outcomes

As noted in a recent brief on the impacts of family planning on nutrition, “undernutrition, which includes stunting, underweight, wasting, and vitamin deficiencies, contributes to nearly half of all childhood deaths. This means that about 3.1 million children under age 5 die each year from malnutrition-related causes.”^13^

The breastfeeding method of family planning—the Lactational Amenorrhea Method (LAM), considered a modern method of family planning^14^—yields all of the nutritional benefits of exclusive breastfeeding, and thus can directly influence newborn and infant nutritional status. However, correct use of this method globally is low at 26% of reported LAM users.^15^ Scaling up correct LAM use globally could bring tremendous nutritional benefits to newborns and infants and prevent unwanted pregnancy among postpartum women for 6 months, before transitioning to another modern method.

Family planning also helps women time and space their pregnancies to ensure healthy nutritional outcomes:

- Spacing pregnancies at least 24 months apart (the equivalent of 3 years between births) is linked to reduction of a key measure of malnutrition—stunting—among children under 5. Children born after a 2-year interval or less, compared with a 4-year interval, are 27% more likely to be stunted and 23% more likely to be underweight.^16^
- Timing pregnancy to occur after age 18 improves adolescents’ growth and development^17^^,^^18^ and reduces the risk of poor outcomes for their children—stunting, low birth weight, and preterm birth.^19^
- Spacing pregnancies helps women replenish essential nutrients. Studies have found that “strong evidence” exists for women’s folate depletion at 3 to 12 months postpartum,^21^ a deficiency linked to the risk of low birth weight in the next pregnancy.^22^^,^^23^
- Spacing pregnancies also gives mothers more time, energy, and resources to breastfeed their infants. And when pregnancies are planned, research shows that mothers can breastfeed for longer periods of time and breastfeeding practices improve, leading to improved nutrition.^13^^,^^24^^,^^25^^,^^26^

Family planning helps women time and space their pregnancies to ensure healthy nutritional outcomes.

**Figure:**
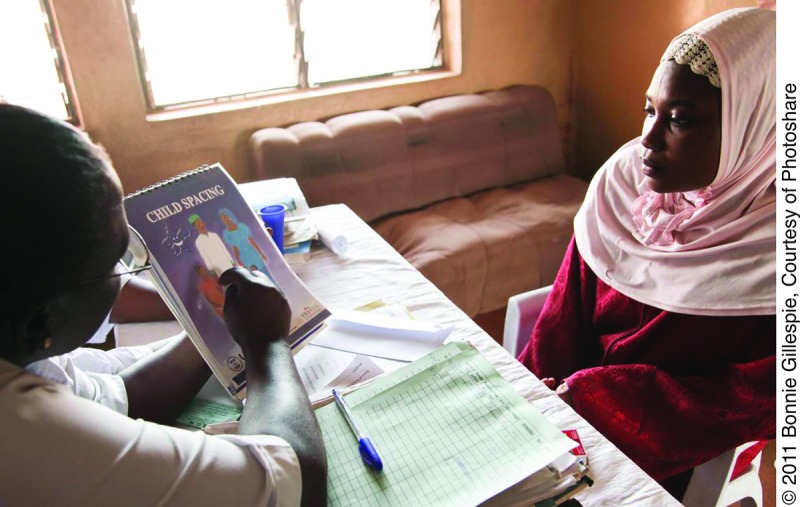
A provider counsels a young woman on birth spacing at a primary health care center in Kagoro, Nigeria.

### Goal 3. Good Health and Well-Being: Ensure Healthy Lives and Promote Well-Being at All Ages

#### Family Planning Saves Lives

Every day, approximately 830 women die from causes related to pregnancy and childbirth. Nearly all—99%— of these maternal deaths occur in low-income countries. More than half of the deaths occur in sub-Saharan Africa, while one-third occur in South Asia. In addition, in 2015 5.9 million children died who were under 5 years of age.^27^

Analyses indicate that, between 2012 and 2020, family planning could help avert approximately 7 million under-5 deaths and prevent 450,000 maternal deaths in 22 priority countries of the U.S. Agency for International Development (USAID).^28^ A modeling study of 172 countries estimated that, in 2008 alone, family planning averted 272,040 maternal deaths—a 44% reduction compared with the maternal deaths that would have occurred without contraceptive use.^29^ It also estimated that satisfying unmet need for contraception could prevent another 104,000 maternal deaths per year (an additional 30% reduction).

Demographic high-risk pregnancies—pregnancies that occur too early or late in the mother’s age, are too closely spaced, or are considered too many (high parity)—are associated with higher risk of mortality or morbidity. Family planning improves the health of women and children by reducing the *proportion* of pregnancies that are considered to be high risk. Family planning also reduces the number of women exposed to pregnancy-related health risks, thus lowering the *number* of unintended pregnancies and births.

The global community generally agrees that family planning prevents maternal deaths by:

- Reducing the number of times a woman is exposed to the risks of pregnancy^30^^,^^31^
- Helping women avoid unintended and closely spaced pregnancies—a study in Bangladesh found that very short pregnancy intervals are linked with 7 times increased risk of induced abortion^32^
- Helping women avoid more than 4 births, or births after 35 years of age^30^

The healthiest times for a pregnancy are between the ages of 18 and 34 and at least 24 months after a birth (which ensures about 3 years between births), while avoiding more than 4 births.

The healthiest times for a pregnancy are between the ages of 18 and 34 and at least 24 months after a birth while avoiding more than 4 births.

On newborn and child health, a wealth of studies conducted in both rich and poor countries, using diverse data sets, have found that spacing pregnancies at least 24 months after a live birth (or about 3 years between births) is associated with lower newborn, infant, and child mortality.^23^^,^^33^^,^^34^^,^^35^ Other studies, focusing on contraceptives, despite mixed results have concluded that family planning helps women space their births and “is protective against short intervals.”^36^ Current analyses indicate that spacing births reduces the risk of death in infancy by up to 10%, and for children under age 5 by 21%.^16^^,^^31^^,^^35^

Questions remain on family planning’s effects on children after they are born. A recent study among infants in Kenya found that a preceding birth interval of less than 18 months was associated with a twofold increase in mortality risk (compared with a birth interval of 36 months), while succeeding intervals of less than 20 months were associated with a 245% increase in early childhood mortality, compared with last births.^20^ In another study, children in Afghanistan with a preceding birth interval less than 18 months or greater than 60 months had significantly higher risks of dying due to diarrhea, sepsis, and low birth weight than children with a preceding birth interval of 24–35 months.^37^ Finally, a systematic review found evidence for folate depletion, vertical transmission of infection, and transmission of infectious disease between siblings as mechanisms that may explain the adverse perinatal, infant, and child health outcomes associated with short intervals.^22^

Questions also remain on the role of family planning *programs* in child survival. A recent analysis of trends in 57 countries (1985–2013) in the modern contraceptive prevalence rate (mCPR) and high-risk births found that the countries with the fastest mCPR progress experienced the greatest declines in high-risk births, including those due to short birth intervals, high parity, and older-age births. The analysis found no significant change in births to women younger than 18 years of age, and it did not examine first births. It also found that 63% of the increase in the mCPR was due to family planning program efforts, 21% due to economic development, and 17% due to women’s education.^38^

Family planning helps women bear children at the healthiest times of their lives—when they are psychologically, physically, emotionally, and economically ready for a pregnancy and thus most likely to survive, stay healthy, and have healthy children. Through strengthened, integrated service delivery and improved counseling for women and girls, especially on the risks of short birth intervals,^39^^,^^40^^,^^41^ high parity, and advanced maternal-age pregnancies,^42^ family planning should be playing a larger role in child and maternal survival and in adolescent health and well-being.

#### Family Planning Prevents HIV/AIDS Transmission

In an era when approximately 34 million adults and children are living with HIV/AIDS, and women of childbearing age account for nearly half of the infected population, family planning has a critical role to play in curbing the HIV/AIDS epidemic.

Family planning has a critical role to play in curbing the HIV/AIDS epidemic.

Correct and consistent use of male or female condoms prevents transmission of the HIV virus. It also prevents unintended pregnancy in women with HIV, and thus potential transmission of the virus to the newborn, as well as maternal deaths (including those related to HIV). A modeling study found that, in the 14 countries with the largest numbers of pregnant women with HIV (at the time of the study), programs to prevent perinatal HIV transmission would prevent over 240,000 infant HIV infections if all women in need used the most efficacious antiretroviral regimen available; the estimated cost would be over US$131 million, or US$543 per infant infection averted per year. In comparison, the annual cost of providing family planning to all women with HIV who wished to prevent unintended pregnancies was estimated at about US$26 million in the 14 countries (US$33 million globally). If all unmet needs for family planning were satisfied for pregnant women with HIV, 423,000 births could be prevented at a cost of US$61 per birth averted in the 14 countries.^43^

While approximately 1 in 4 women in sub-Saharan Africa has an unmet need for family planning, studies have shown that women living with HIV have higher unmet need for family planning and reproductive health services than the general population, in part due to lack of investment in integrated family planning and HIV services.^44^ For example, a 2012 study found that programs that have “succeeded in promoting condom use and providing HIV prevention and treatment services … have largely missed the opportunity to address the contraceptive needs of the key populations they serve.”^44^ Another recent study found that if the needs of women with HIV for modern contraceptive methods and antiretroviral medication were both fully met, HIV transmission from mothers to newborns would be nearly eliminated—reduced by 93% annually,^5^ greatly contributing to the “AIDS-Free Generation” goal of the U.S. President’s Emergency Plan for AIDS Relief (PEPFAR). Pursuing opportunities to advance family planning integration with HIV services would significantly expand family planning access across Africa and address unmet need.

### Goal 4. Quality Education: Ensure Inclusive and Equitable Quality Education and Promote Lifelong Learning Opportunities for All

#### Family Planning Supports Women’s and Girls’ Education

Family planning can help women and girls, especially those who have become mothers, stay in school, become literate, learn a trade, start a business, or otherwise achieve their educational and employment goals. Early and unintended pregnancy can be both a cause and a consequence of dropping out of school.^45^

Early and unintended pregnancy can be both a cause and a consequence of dropping out of school.

Since 2000, adolescent pregnancy has declined only modestly in most countries,^46^ and adolescents continue to face many barriers in obtaining contraceptive services and commodities.^47^ In 2015, in 56 USAID-assisted countries, approximately 22 million adolescents ages 15–19 had begun childbearing and, of these, 4.3 million have had a second or third child.^48^ Adolescent pregnancy affects the adolescents themselves, their families, communities, and broader society.^18^ With such high numbers of adolescent pregnancies globally, the world “squanders the well-being, talents, and contributions” of the 20,000 young girls under the age of 18 who give birth each day.”^49^

Research shows how family planning supports girls’ and women’s education:

- An analysis in Iran explored married women’s contraceptive use and education and found that those using a modern contraceptive method before the first birth were 84% more likely to advance their education by 1 to 2 years than those not using any method before the first birth.^50^
- An analysis of 200,000 married women from 242 districts in 26 African countries concluded that the number of births to women with children under age 6 and short intervals between the last 2 children “have substantial negative effects” on women’s employment outside agriculture.”^51^
- A series of case studies concluded that “overall well-being of women and girls improves as fertility declines, especially as it relates to their maternal health, educational attainment, and workforce participation,” and fertility decline has had a more positive impact on girls’ education than it has had on boys’ education.^52^
- A study in Bangladesh found wide-ranging and multiple, positive impacts of family planning on the education and empowerment of women and girls. Women in the family planning-maternal and child health intervention area had not only fewer children with longer intervals between births but also higher individual and household incomes than that of the women and households in the comparison group. The daughters of the program households were better educated than the daughters of families who were not in the program.^53^
- The lifetime opportunity costs of adolescent pregnancy—a measure of the annual income adolescent mothers forgo over their lifetime—range from 1% of annual gross domestic product (GDP) in a large country, such as China, to 30% of annual GDP in a smaller economy such as Uganda. If adolescent girls in Brazil and India were able to wait until their early twenties to have children, the increased economic productivity would equal more than US$3.5 billion and US$7.7 billion, respectively.^54^

### Goal 5. Gender Equality: Achieve Gender Equality and Empower All Women and Girls

#### Family Planning Advances Gender Equality and Empowerment

Gender equality and empowerment call for equal access to resources, services, and opportunities. Gender equality refers to equal enjoyment of human rights, goods, opportunities, and services among women and men, while empowerment refers to expanding people’s capacity to make and act on decisions.^55^ They require addressing the barriers women face in making decisions about their own daily lives.^46^ Women’s access to their chosen family planning method and their ability to negotiate use of the method, therefore, strongly supports gender equality and empowerment.

Women’s access to their chosen family planning method and their ability to negotiate use of the method strongly supports gender equality and empowerment.

Many women, however, are unable to make and act on decisions affecting their reproductive lives. A 2014 report found that less than half of currently married women use modern contraception in 37 of 46 countries, and around one-quarter or more of currently married women have an unmet need for family planning in 21 of the 46 countries.^46^ High levels of unmet need may indicate that women are not empowered to use contraception because they lack access to health care or are unable to negotiate family planning with their partner. Increasing women’s ability to choose the number, timing, and spacing of their children, or their ability to decide if they want to bear children at all, is fundamental for women’s control over the circumstances of their lives and for the full achievement of SDG 5.

While family planning programs are not the only contributors to increasing equality, empowerment, and education, the evidence is clear that family planning makes a critical contribution toward achieving these global goals. These broader societal impacts have been achieved, in part, through well-designed and implemented service delivery programs that reach underserved communities—programs that should now be scaled up across Africa and Asia.^56^^,^^57^^,^^58^^,^^59^^,^^60^

## PLANET

In 2016, scientists issued an urgent environmental call—“we have a global emergency”^61^—and predicted that climate change will be quicker and more catastrophic than anticipated.^62^ They stated that even 2 degrees Celsius^63^ of global warming would be too much and recommended that “fossil fuel CO_2_ emissions should be reduced as rapidly as practical.” While not all agree with the scientists’ dire assessment, their pragmatic recommendations are ones that all concerned with planetary health should heed. Family planning has a critical role to play in the growing social movement of support for the transformation from “public to planetary health.”^64^

Family planning has a critical role to play in the planetary health movement.

Clear and compelling evidence points to serious consequences of rapid population growth on environmental outcomes. Population dynamics, including human population size, growth, density, and migration, are important drivers of environmental and natural resource degradation, including land, forests, biodiversity, and water.^65^ The relationships are complex, mediated by poverty, technology, and management practices, among other factors.^66^ However, as recognized in the 2013 Second International Population, Health, and Environment Conference, “poor reproductive health outcomes and population growth exist hand-in-hand with poverty and unsustainable natural resource use,” especially in remote and rural communities.^67^ Although up-to-date empirical data on the specific role of family planning is scarce, a recent review of the existing evidence found that integrating family planning into non-health sector projects, such as natural resource management, has led to improvements in environmental indicators, increased use of contraceptives, and, in instances where long-term measurement was possible, declines in parity or crude birth rates.^67^

### Goal 6. Clean Water and Sanitation: Ensure Availability and Sustainable Management of Water and Sanitation for All

#### Family Planning Mitigates Population Growth’s Effects on Access to Water and Sanitation

In 2014, the World Economic Forum identified water crises as the global systemic risk of third highest concern.^68^ Population growth affects water scarcity in important ways. It contributes to increased demand and competition for water for domestic, industrial, and municipal uses, including irrigation, and limits the amount of water available per person. Estimates suggest that by 2035, 3.6 billion people will be living in areas of water stress or scarcity, up from approximately 2 billion today.^69^

Population growth also negatively affects access to sanitation. The United Nations Children’s Fund (UNICEF) and the World Health Organization (WHO) estimate that, in 2015, some 2.4 billion people—over one-third of the world’s population—lacked access to improved sanitation.^70^ Between 1990 and 2011, Eastern and Southern Africa and West and Central Africa experienced massive population growth, and the number of people practicing open defecation in both regions rose to over 100 million.^70^ In sub-Saharan Africa overall, the number of people defecating in the open is still increasing, largely due to population growth, declared UNICEF and WHO.^70^

To what extent can family planning influence availability of water and sanitation? While the evidence is not extensive:

- Analyses have highlighted water availability under projected scenarios of high and low fertility. For example, estimates suggest that in Jordan, with low fertility (total fertility rate of 2.1 rather than the current rate of 3.8) 26% less water (644 cubic meters versus 733 cubic meters) would be required for the country as a whole in 2040.^71^
- Analyses have also found that family planning programs in Egypt and Jordan have generated modest sectoral savings in water and sanitation (but significant sectoral savings in health and education).^71^^,^^72^

### Goal 7. Affordable and Clean Energy: Ensure Access to Affordable, Reliable, Sustainable, and Modern Energy for All

#### Integrated Population, Health, and Environment Projects Can Expand Access to Clean and Renewable Energy

Access to clean and renewable energy is a global issue. Clean energy is defined as heat and electricity produced from renewable sources (wind, sun, rain, waves, tides), generating little or no pollution or emissions. In contrast, approximately 2.8 billion people cook with firewood and other fuels that are linked with health issues and widespread deforestation.^73^ In 2012, at least 4.3 million premature deaths, mostly to women and children, were attributed to household air pollution and the effects of reliance on polluting cook stoves.^74^

Over the years, population growth has eroded renewable energy gains. A 2013 World Bank report found that although 1.7 billion people gained access to electricity in the last 10 years, “this is only slightly ahead of population growth of 1.6 billion over the same period.”^73^ Therefore, “the pace of expansion will have to double” to meet the 2030 targets for modern electricity access.

Integrated population, health, and environment (PHE) projects have successfully introduced both family planning and clean energy into communities. A family planning project in Uganda collaborated with a clean energy provider to provide solar lights to family planning peer educators. Prior to the project, the peer discussions were necessarily held during daylight hours and thus engaged women only. The solar lights, powered by the sun, were cleaner, more sustainable, and less expensive than kerosene, and they enabled the peer educators to visit homes at night—when the men would be home from work—to talk to couples about family planning.^75^

Integrated population, health, and environment projects have successfully introduced both family planning and clean energy into communities.

### Goal 9. Industry, Innovation, and Infrastructure: Build Resilient Infrastructure, Promote Inclusive and Sustainable Industrialization, and Foster Innovation

#### Family Planning Contributes to Building Resilient Infrastructures

Resilience refers to the ability of households, communities, systems, and countries to respond to and recover from shocks and stresses in ways that reduce chronic vulnerability and facilitate inclusive growth.^76^

The dramatic case of the Sahel demonstrates the important role that family planning can play in helping to create resilient countries and communities. Since 1960, the Sahel, “one of the most chronically vulnerable regions of the world”^76^ encompassing 10 countries and 100 million inhabitants, has experienced severe drought, food insecurity, low rainfall, environmental degradation, and civil conflict, leading to declining agricultural production. All countries in the Sahel have experienced the “gendered nature of natural disasters.”^77^ Four of the 10 countries with the highest total fertility rates in the world are in the Sahel (Niger 7.6, Chad 6.5, Burkina Faso 6.0, and Mali 5.9).^76^ Contraceptive use by married women is extremely low—for example, less than 2% of married women in Chad use contraception.

Continuing this trajectory of high fertility and low contraceptive use will severely undermine these countries’ abilities to respond to social sector needs. In Niger, population growth is 4% annually and will double in just 20 years. “This growth will require a massive investment in schools, health clinics, and job creation for youth,” with additional investment also needed in agriculture and livestock systems to ensure food security. Increased investment in family planning in the region could make a critical step toward resilience.^78^

An analysis in Egypt helps us understand how family planning and lower population growth helped build a more resilient infrastructure for health and economic development. The Egyptian family planning program contributed to a decline in the total fertility rate, from 5.6 in 1976 to 3.1 in 2005. During this time, use of contraception increased from 19% to 59%, made available through the expansion of public-sector and NGO clinics.^72^ Under conditions of constant fertility in Egypt, by 2040 there would be 3.6 million births per year. On the other hand, if the fertility rate were lowered, by 2040 there would be about 2 million births per year (about 2.4 per woman). Under this scenario, there would also be^79^:

- 1.3 million fewer people entering the labor force
- 6.4 million fewer primary students in 2040
- 80 billion Egyptian Pounds saved in health care costs
- 20% more land per person in 2040
- 13% less water required
- 17% more electricity available per person

Family planning and lower population growth can help build a more resilient infrastructure for health and economic development.

Analysis revealed that, between 1980 and 2005, the family planning program contributed to 45,838 million Egyptian Pounds in savings in expenditures on education, child health, and food subsidies, while costing 2,402 million Egyptian Pounds. The resulting lower health care and education costs, and more land, water, electricity, and jobs available per person, has yielded multiple development benefits for Egypt and an improved quality of life for Egyptians.^72^

### Goal 11. Sustainable Cities and Communities: Make Cities and Human Settlements Inclusive, Safe, Resilient, and Sustainable

#### Family Planning Contributes to Building Safe, Resilient, and Sustainable Cities

Studies have found that it is “not only rapid population growth, but rapid urbanization that is causing problems for the poorest countries.”^80^ Estimates indicate that the world is confronting the largest wave of urbanization in human history.^81^ By 2030, about 5 billion people will live in cities,^81^ putting huge pressure on infrastructures, such as health, water, sanitation, and education.

- The urban population in 2014 accounted for 54% of the total global population, up from 34% in 1960, and continues to grow.^82^
- Africa is experiencing the most rapid urbanization in the world, with annual urban growth rates in recent years of 3.36% per year.^83^ As the UN Economic Commission for Africa stated in a recent report, “Urbanization, together with Africa’s approaching demographic transition, may well become the most decisive determinants of Africa’s economic and social development since independence.”^83^
- The estimated number of slum dwellers is increasing, from over 650 million in 1990 to about 863 million in 2012.^81^ In Africa between 1990 and 2010, the proportion of urban residents living in slums declined from 70% to 62%, “yet the actual number of slum dwellers has almost doubled from 103 million to 200 million.”^84^

**Figure:**
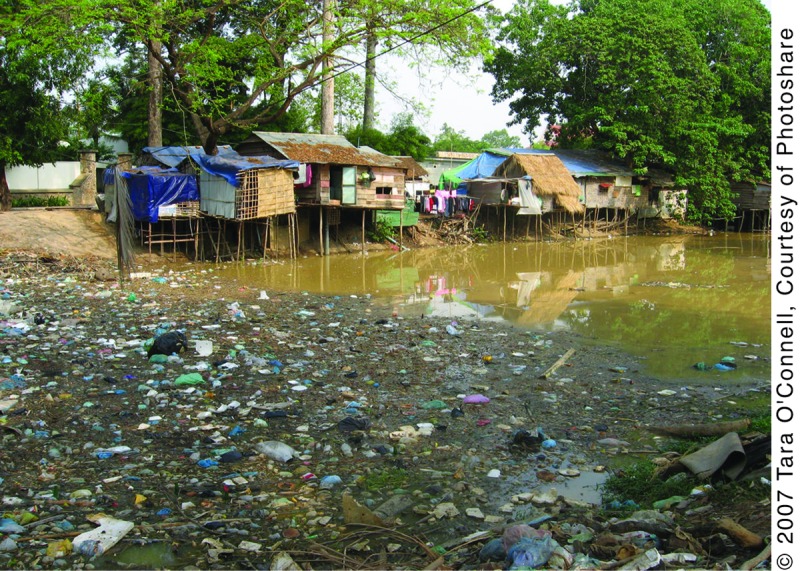
Houses in Cambodia stand on the Siem Reap River, which is clogged with litter and refuse.

Urban growth in Africa is happening so quickly that it is overwhelming governments’ abilities to provide education, health services, housing, drinking water, transportation, electricity, and waste disposal.^84^ The latest Demographic and Health Survey data show that although urban women in Africa continue to have fewer children than their rural counterparts,^85^ the urban total fertility rate is still above 3 in most countries.^85^ While many assume that urban dwellers have greater access to family planning messages and services than rural populations, in many instances this is not the case.^86^

Findings from a longitudinal family planning study in the urban areas of India (Uttar Pradesh), Kenya, Nigeria, and Senegal shed light on effective, evidence-based strategies to address the unmet need for family planning of urban women and slum dwellers. The study found that exposure to demand-generation activities was significantly and positively associated with modern method use in all of the countries’ studied, showing that “targeted, multi-level demand generation activities can make an important contribution to increasing modern method use in urban areas of Africa.”^87^

### Goal 12. Responsible Consumption and Production: Ensure Sustainable Consumption and Production Patterns

#### Family Planning Can Help Reduce Population Effects on Food and Chemical Waste

According to the United Nations Environment Programme, “The well-being of humanity, the environment, and the functioning of the economy, ultimately depend upon the responsible management of the planet’s finite natural resources. These challenges are mounting as the world population is forecast to reach over 9 billion by 2050.”^88^ Sustainable consumption and production is about managing finite resources and energy efficiency. Key SDG 12 targets involve managing resources efficiently by reducing waste—for example, by 2030, halving per capita food waste, reducing waste generation, and achieving sound management of chemical waste.

Countries of the Organisation for Economic Co-operation and Development (OECD) produce almost half of the world’s waste, while Africa and South Asia produce the least. But the issue of waste management is relevant for Asia and Africa and for family planning because, as a World Bank report observed, “… waste is inextricably linked to urbanization.”^89^ It noted, “Today, more than 50% of the world’s population lives in cities, and the rate of urbanization is increasing quickly.” As urbanization increases, income and consumption also increase, leading to a corresponding increase in waste. The report found^89^:

- Improving waste management in low-income countries is an urgent priority.
- Poor waste management has an enormous impact on health and well-being—contributing to flooding, pollution, respiratory ailments, diarrhea, and dengue fever.
- Today there are about 3 billion urban residents generating about 1.2 kg of waste per person per day; by 2025, this will increase to 4.3 billion urban residents generating about 1.4 kg of waste per person per day.
- In lower-income cities, solid waste management is usually the single largest budgetary item.

Given the urgency of these issues, studies are needed on the role family planning could play as it relates to urbanization and reduction of food and chemical waste, and the sectoral financial savings that might be generated as a result.

### Goal 13. Climate Action: Take Urgent Action to Combat Climate Change and Its Impact

#### Family Planning Helps Address the Challenges of Climate Change

Population dynamics have an important connection to both the challenges of and solutions to the problem of climate change. Rapid population growth exacerbates vulnerability to the negative consequences of climate change and exposes growing numbers of people to climate risk. The Intergovernmental Panel on Climate Change (IPCC) considers population (along with economic growth and technical change) “one of the root causes of greenhouse gas emissions.”^90^ Meeting family planning needs will stem population growth, easing challenges associated with adapting to climate change impacts and reducing the growth of greenhouse gas emissions.

- A 2015 study on family planning as a cost-effective strategy to address food insecurity and climate change concluded that slowing population growth can “slow global climate change, by providing 16% to 29% of the needed emissions reductions” by 2050 and reduce the need to increase food production.^91^^,^^92^ By the end of the century, the effect of slower population growth could reduce total emissions from fossil fuel use by 37% to 41%.
- Another study found that improving access to family planning is a relatively inexpensive intervention for reducing carbon emissions compared with other strategies such as solar, wind, and nuclear power; biofuels; or carbon capture and storage.^93^
- The IPCC argues that “providing access to reproductive health services (including modern family planning)” is an opportunity “to achieve co-benefits … to improve child and maternal health through birth spacing and reduce population growth, energy use, and consequent CAP [climate-altering pollutants] emissions over time.”^94^
- An analysis examining the relationship between food security, population growth, and climate change in Ethiopia showed the potential of family planning to address the food security gap resulting from decreased crop productivity due to climate change. Assuming the current pace of climate change continues, the study found that, by the year 2050, slower population growth would “compensate completely for the effects of climate change on food insecurity.”^95^

Population dynamics have an important connection to both the challenges of and solutions to the problem of climate change.

An analysis found that between 2004 and 2009, in government reports articulating priorities for climate change adaptation, 37 of 40 governments recognized that population growth was important for climate change, yet only 6 proposed activities to address it.^96^ The analysis called for broad-based adaptation of an integrated approach and gave the example of an integrated watershed management project in Ethiopia in Wichi province that aimed to improve crop production, minimize biodiversity loss, and increase access to family planning and HIV/AIDS awareness. It concluded that governments’ repeated emphasis on the relevance of demographic trends in their climate change adaptation plans “provide a strong collective case for the ‘mainstreaming’ of an integrated approach … exemplified by the Ethiopian case study.” Such a call is still highly relevant today.

### Goal 14. Life Below Water: Conserve and Sustainably Use the Oceans, Seas, and Marine Resources for Sustainable Development

#### Family Planning Helps to Protect Declining Marine Resources

Under intense population pressure, global fisheries are disappearing and ocean resources are becoming extinct.

- A 2009 study of global commercial fisheries found that “80% of fish stocks have either been fully exploited, overexploited, or have collapsed.”^97^ While reducing the catch by 20% to 50% is needed for sustainable fishing, demand for fish is expected to increase by 35 million tons due to increased consumption and population.
- Of the 21 marine species known to have become extinct in the past 300 years, 16 disappeared since 1972.^98^ Population growth affects the oceans in many ways including coral reef damage; accidental killing of millions of tons of birds, fish, and sea turtles; runoff laced with massive chemical fertilizer applications creating ocean “dead zones;” and a vast amount of discarded waste of 6.8 billion consumers, which finds its way to the oceans.
- Population growth will likely impact the success of programs meant to help species rebound and protect the ocean ecosystem. More research is needed on how family planning can support the protection of oceans and marine resources.

### Goal 15. Life on Land: Protect, Restore, and Promote Sustainable Use of Terrestrial Ecosystems, Sustainably Manage Forests, Combat Desertification, and Halt and Reverse Land Degradation and Halt Biodiversity Loss

#### Family Planning Helps Mitigate the Effects of Deforestation and Unhealthy Interaction Among Humans, Domestic Animals, and Wildlife

The world loses approximately 14.5 million hectares of forest each year.^99^ As populations grow rapidly, the demand for food and forest products also grows, and forest areas are turned into fields for agriculture and commercial forestry.

As populations grow rapidly, the demand for food and forest products also grows.

A comprehensive study of 46 countries in Africa, Asia, and Latin America found that “agriculture is the main driver of deforestation, causing 73% of all deforestation.”^100^ Deforestation threatens the well-being and livelihoods of millions of people who heavily depend on forest resources.^101^^,^^102^ Deforestation also contributes to biodiversity loss: it is estimated that the Southeast Asia region, which has the highest relative rates of deforestation, will lose three-quarters of its original forests and up to 42% of its biodiversity by 2100.^103^ Rapid population growth is a driver of biodiversity loss.

Preventing desertification and land degradation is also part of these goals. Desertification occurs with intensive farming, as well as changing climate conditions. Population density contributes to soil depletion and erosion. Providing men and women with family planning to achieve their desires for smaller family sizes will contribute to reduced rates of deforestation, desertification, and land degradation.

Population dynamics can also contribute to unhealthy interactions among humans, domestic animals, and wildlife. Human population density has been found to be a “significant independent predictor” of emerging infectious diseases.^104^ Population expansion is linked to other underlying drivers of disease emergence, including environmental changes. For example, increasing interaction among humans, domestic animals, and wildlife, following land use change, is considered to be a significant contributor to disease emergence.^105^

## PROSPERITY

### Goal 8. Decent Work and Economic Growth: Promote Sustained, Inclusive, and Sustainable Economic Growth, Full and Productive Employment, and Decent Work for All

#### Family Planning Contributes to Economic Growth

One important way that family planning contributes to economic growth is by facilitating changes in a country’s age structure. Rapid fertility decline, which is linked to increased family planning use, lowers the ratio of dependents to income earners. This results in a higher proportion of wage earners and leads to national savings. With supportive socioeconomic policies and attention to equity, countries can then experience a “demographic dividend” of rapid economic growth. Estimates indicate that the demographic dividend effect of family planning is most pronounced in countries with current high fertility, where rates of return on economic productivity and potential lifetime earnings from improved availability and uptake of contraception could exceed 8% of GDP by 2035.”^1^

In the case of the East Asian Tigers (Hong Kong, Singapore, South Korea, and Taiwan), the demographic dividend lasted up to 25 years and has been estimated to account for between 25% to 40% of East Asia’s “economic miracle.”^106^ Across Africa, estimates suggest that a demographic dividend could raise average incomes by 56% compared with a scenario in which the share of the working age population remains constant.^107^ The demographic dividend is a window of opportunity for countries to take advantage of a robustly expanding workforce. The payoffs will be high if social and economic policies support the education and employment of young people, especially girls.

South Korea and Thailand, demographic dividend success stories, represent strong examples of countries’ aligning population policy and family planning services with human capital development policies to accelerate economic growth.^108^^,^^109^^,^^110^

## PEACE

As a multi-sectoral intervention, family planning also contributes to reaching vulnerable populations, mitigating conflict, and achieving state stability and peace.

### Goal 10. Reduced Inequalities: Reduce Inequality Within and Among Countries

#### Family Planning Promotes Inclusive Societies by Addressing the Needs of Disadvantaged Populations

SDG 10 states, “There is growing consensus that economic growth is not sufficient to reduce poverty if it is not inclusive. … To reduce inequality, policies should be universal in principle paying attention to the needs of disadvantaged and marginalized populations.”^111^

Unmet need for contraception is often highest among the most disadvantaged and vulnerable—adolescents, the poor, those living in rural areas and urban slums, people living with HIV, and internally displaced persons. These groups have the fewest resources and are the least able to deal with the demands of an unexpected pregnancy. Postpartum women have especially high unmet need: 61% of women within 1 year of their last birth have an unmet need for modern contraceptive methods.^112^ Effective family planning programs reach these underserved populations and will need to accelerate efforts in this area if the universal access goals of SDGs 3.7 (universal access to sexual and reproductive health-care services), 5.6 (universal access to sexual and reproductive health and reproductive rights), and 10 (reduce inequality) are to be achieved. A 2015 study showed that, overall, the poor-rich gap in contraceptive use is diminishing, and even more so when family planning programs are strong. Gaps remain in many sub-Saharan African countries.^113^

The poor-rich gap in contraceptive use is diminishing, but gaps remain in many sub-Saharan African countries.

At the individual and household level, experts note that identifying the effect of demographic factors on economic welfare has “proved elusive,” and finding the links between household poverty and childbearing has “proved contentious.”^8^ As discussed under the SDGs for poverty, education, and equality, some studies show that more women are likely to enter the labor force with fewer children^114^; families who received family planning and maternal-child health services were more likely to have higher incomes and greater savings and assets^53^^,^^115^; and fewer children per family leads to increased household savings and increased investments in each child.^116^ A study from Pakistan found that the direct effect of more children of all age/sex combinations on savings is negative and substantial,^117^ and a study from Nigeria found that household size was linked with the probability of being poor.^118^

These few studies suggest that new research is needed on fertility’s effects on household income and savings.

### Goal 16. Peace, Justice, and Strong Institutions: Promote Peaceful and Inclusive Societies for Sustainable Development, Provide Access to Justice for All, and Build Effective, Accountable, and Inclusive Institutions at All Levels

#### Family Planning Contributes to Peace and Stability

Studies have shown that a large “youth bulge” (defined as a high proportion of youth 15 to 29 years old relative to the older adult population) is associated with a high risk of civil conflict.^119^ That is, states with youthful age structures—especially within a politically organized minority^120^—are more likely to experience armed, intrastate conflict and other types of violence.

The political impact of fertility decline is significant. As a country and its population age, studies show that the probability of attaining and maintaining a liberal democracy is increased.^121^

Currently, more than 40 countries are “young,” with total fertility rates above 4 children per woman. However, in another 70 countries the demographic transition is more advanced, and the chances for liberalization—and stability—are greater.^122^

In many of the “young” countries, large numbers of alienated youth cannot find jobs and are easy recruits for radical groups that can provide a regular salary. While family planning is not the sole solution, it is important to understand that the high proportion of jobless youth relates to the overall population structure and the sluggish economies that cannot support even menial jobs for everyone. Other experts also see the “youth bulge” as a possible precursor to violence. They urge such countries to consider increasing support for girls’ education, family planning, and youth employment, contending that “the pill is mightier than the sword.”^123^

**Figure:**
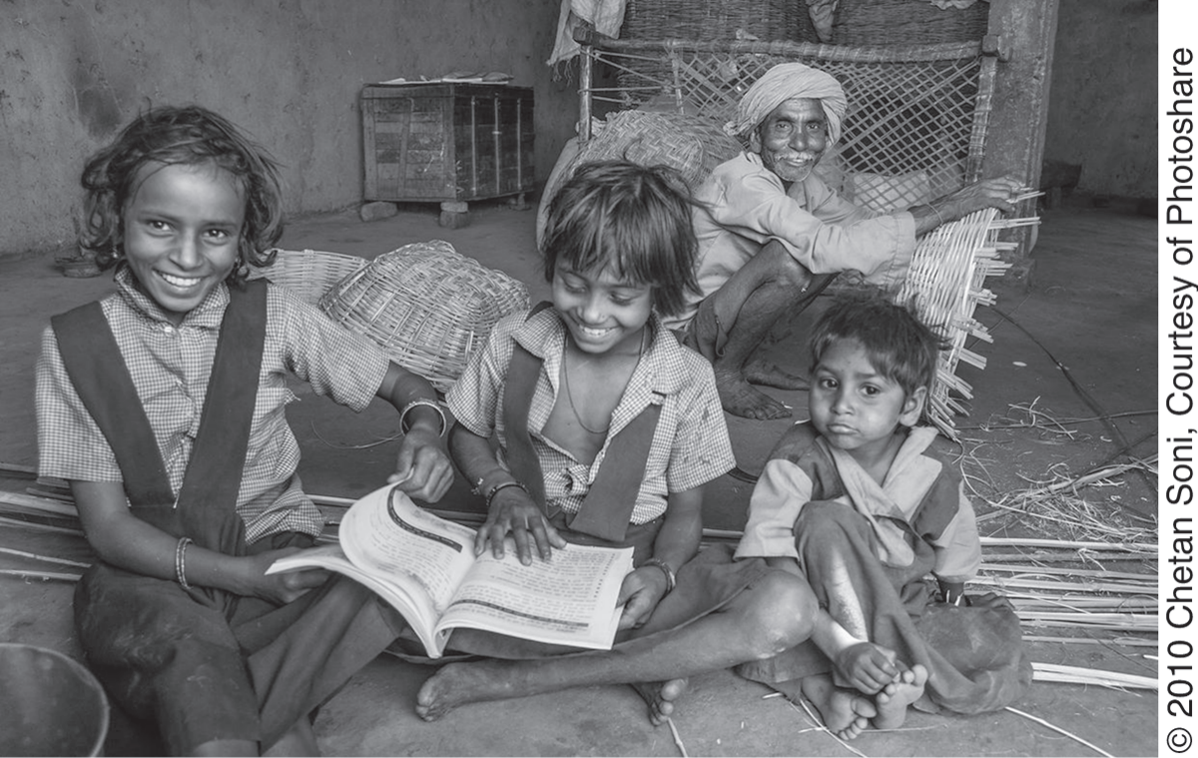
With the help of an NGO in Alirajpur district, India, a father started a basket-weaving business, allowing him to send his girl child to school.

## PARTNERSHIP

### Goal 17. Partnerships for the Goals: Strengthen the Means of Implementation and Revitalize the Global Partnership for Sustainable Development

#### Family Planning Partnerships Can Support the Achievement of the SDGs

A revitalized family planning agenda continues to be needed.^8^ Family planning services still fall short of need in all developing regions, though analyses show that for every dollar invested in family planning, between US$1.47 and US$4.00 is saved in maternal and newborn health care.^5^^,^^124^ Investing in family planning, in addition to maternal and newborn services, can save US$1.5 billion while achieving the same health outcomes.^5^ According to the new Global Investment Framework for Women’s and Children’s Health, scaling up access to and use of modern contraceptive methods would directly avert 53% (78 million) of the 147 million child deaths prevented under the high-investment scenario but would require only 4% of intervention-specific costs between 2015 and 2035.^1^

For every US$1 invested in family planning, up to US$4 is saved in maternal and newborn health care.

A wide range of global partnerships have made important strides in recent years in promoting and strengthening family planning. These partnerships—including FP2020, the UN Commission on Life-Saving Commodities, the Ouagadougou Partnership, and the MDG Health Alliance—provide a foundation and a model for the collaborative, multi-sectoral efforts that are needed to support sustainable development. Moreover, these partnerships will be critical in supporting country-level partnerships that include the public and private commercial sectors, foundations, civil society organizations, and non-health sector groups (e.g., in education, environment, income generation) to accelerate country-level change in the years ahead, and to ultimately achieve the Sustainable Development Goals.

## CONCLUSION: INVEST IN FAMILY PLANNING TO ACHIEVE THE SDGS

In the time frame of the SDGs, the world has the opportunity to achieve a grand convergence between the developed and developing world, ending preventable child and maternal deaths and achieving relative parity in meeting the family planning needs of women, men, couples, and adolescents who want to space or limit childbearing.

Family planning can accelerate progress across the 5 SDG themes of People, Planet, Prosperity, Peace, and Partnership and is critical to achieving the goals and the post-2015 development agenda (see Box for a summary). Empowering women to choose the number, timing, and spacing of their pregnancies is not only a matter of health and human rights but also touches on many multi-sectoral determinants vital to sustainable development, including women’s education and status in society. Without universal access to family planning and reproductive health, the impact and effectiveness of other interventions will be less, will cost more, and will take longer to achieve. Global strategies and partnerships—and health decision makers at all levels—must leverage the abundance of available research, evidence, and the range of justifications presented here to prioritize family planning as a foundational component of health, rights, and long-term development strategies.

Without universal access to family planning and reproductive health, the impact and effectiveness of other interventions will be less, will cost more, and will take longer to achieve.

BOX **The Central Role of Family Planning in Achieving the Sustainable Development Goals Across the 5 Themes of People, Planet, Prosperity, Peace, and Partnership**

**PEOPLE**

- Family planning advances human rights.
- Family planning helps reduce poverty.
- Family planning contributes to improved nutrition outcomes.
- Family planning saves lives.
- Family planning prevents HIV/AIDS transmission.
- Family planning supports women’s and girls’ education.
- Family planning advances gender equality and empowerment.

**PLANET**

- Family planning mitigates population growth’s effects on access to water and sanitation.
- Integrated population, health, and environment projects can expand access to clean and renewable energy.
- Family planning contributes to building resilient infrastructures.
- Family planning contributes to building safe, resilient, sustainable cities.
- Family planning helps reduce population effects on food and chemical waste.
- Family planning helps address the challenges of climate change.
- Family planning helps to protect declining marine resources.
- Family planning helps mitigate the effects of deforestation and unhealthy interaction among humans, domestic animals, and wildlife.

**PROSPERITY**

- Family planning contributes to economic growth.

**PEACE**

- Family planning promotes inclusive societies by addressing the needs of disadvantaged populations.
- Family planning contributes to peace and stability.

**PARTNERSHIP**

- Family planning partnerships can support the achievement of the SDGs.

## Notes

**Peer Reviewed**

**First Published Online:** 2016 Jun 9
